# Supplementary material for: Approaching national climate targets in China considering the challenge of regional inequality
Source: Nat Commun. 2023 Dec 15;14:8342. doi: 10.1038/s41467-023-44122-0 (PMC10724292; doi:10.1038/s41467-023-44122-0)
Supplement: Supplementary file 1 — Supplementary Information [file 41467_2023_44122_MOESM1_ESM.pdf]

# Approaching national climate targets in China considering the challenge of regional inequality

Biying Yu*, Zihao Zhao, Yi-Ming Wei*, Lan-Cui Liu*, Qingyu Zhao, Shuo Xu,
Jia-Ning Kang, Hua Liao

## Supplementary Tables

**Supplementary Table 1 | Average annual growth rate of national GDP under different scenarios.** LD, MD, and HD denote low demand, medium demand and high demand for products or services in energy-consuming sectors corresponding to different socio-economic development speeds. The GDP growth rate in LD is the lowest and in HD is the highest.

| Scenario | 2021~2025 | 2026~2030 | 2031~2035 | 2036~2040 | 2041~2050 | 2051~2060 |
|---|---|---|---|---|---|---|
| LD | 5.0% | 4.5% | 3.5% | 3.5% | 2.5% | 1.5% |
| MD | 5.6% | 5.5% | 4.5% | 4.5% | 3.4% | 2.4% |
| HD | 6.0% | 5.5% | 5.0% | 5.0% | 4.5% | 4.0% |

**Supplementary Table 2 | Official planning of provinces that have proposed carbon reduction or energy consumption targets.**

| Province | Planned carbon peak time | Other planning |
|---|---|---|
| Tianjin | Before 2025 | - |
| Hebei | Before 2030 | Proportion of non-fossil energy consumption: more than **13%** by 2025 and more than **19%** by 2030 |
| Inner Mongolia | Before 2030 | Proportion of coal consumption: reduce to below **75%** by 2025 |
| Liaoning | Before 2030 | The proportion of non-fossil energy consumption will reach about **20%** in 2030 |
| Jilin | Before 2030 | Proportion of non-fossil energy consumption: about **20%** by 2030 |
| Heilongjiang | Before 2030 | - |
| Shanghai | Before 2025 | - |
| Zhejiang | Before 2025 | - |
| Jiangxi | Before 2030 | Proportion of non-fossil energy consumption: **18.3%** by 2025 |
| Shandong | 2027 | - |
| Henan | Before 2030 | - |
| Hunan | Before 2030 | Proportion of non-fossil energy consumption: **25%** by 2030 |
| Guangdong | Before 2025 | - |
| Guangxi | Before 2030 | Proportion of non-fossil energy consumption: **30%** by 2025, **35%** by 2030 |
| Hainan | Before 2025 | - |
| Chongqing | Before 2030 | - |
| Sichuan | - | Proportion of non-fossil energy consumption: about **66%** by 2030 |
| Guizhou | Before 2030 | - |
| Yunnan | - | Proportion of non-fossil energy consumption: more than **50%** by 2030 |
| Qinghai | Before 2030 | Proportion of non-fossil energy consumption: **80%** by 2030 |
| Ningxia | Before 2030 | Proportion of non-fossil energy consumption: more than **15%** by 2025 and more than **20%** by 2030 |

**Supplementary Table 3 | Upper and lower limits of annual average growth rate of GDP for each province.** This study sets the lower and upper limits of provincial GDP growth rate in future years according to the historical deviation degree from the national GDP growth rate. Future upper limits are obtained from the national GDP under the high-demand (HD) scenario and lower limits are obtained from the low-demand (LD) scenario data.

| | 2023-2025 | | 2026-2030 | | 2031-2035 | | 2036-2040 | | 2041-2050 | | 2051-2060 | |
|---|---|---|---|---|---|---|---|---|---|---|---|---|
| | upper limit | lower limit | upper limit | lower limit | upper limit | lower limit | upper limit | lower limit | upper limit | lower limit | upper limit | lower limit |
| Beijing | 7.1% | 5.9% | 6.5% | 5.3% | 5.9% | 4.1% | 5.9% | 4.1% | 5.4% | 2.5% | 4.9% | 1.2% |
| Tianjin | 2.6% | 2.2% | 2.4% | 2.0% | 2.5% | 1.5% | 2.2% | 1.5% | 2.0% | 0.9% | 1.8% | 0.5% |
| Hebei | 4.4% | 3.7% | 4.0% | 3.3% | 3.7% | 2.6% | 3.7% | 2.6% | 3.4% | 1.6% | 3.1% | 0.8% |
| Shanxi | 4.9% | 4.1% | 4.5% | 3.7% | 4.1% | 2.9% | 4.1% | 2.9% | 3.8% | 1.7% | 3.4% | 0.9% |
| Inner Mongolia | 2.9% | 2.4% | 2.6% | 2.1% | 2.4% | 1.7% | 2.4% | 1.7% | 2.2% | 1.0% | 2.0% | 0.5% |
| Liaoning | 2.4% | 1.5% | 1.9% | 1.6% | 1.9% | 1.2% | 1.7% | 1.2% | 1.6% | 0.7% | 1.4% | 0.4% |
| Jilin | 2.5% | 1.8% | 2.3% | 1.9% | 2.3% | 1.4% | 2.1% | 1.4% | 1.9% | 0.8% | 1.8% | 0.4% |
| Heilongjiang | 2.4% | 1.5% | 1.9% | 1.6% | 1.9% | 1.2% | 1.7% | 1.2% | 1.6% | 0.7% | 1.4% | 0.4% |
| Shanghai | 6.9% | 5.7% | 6.3% | 5.2% | 5.7% | 4.0% | 5.7% | 4.0% | 5.2% | 2.4% | 4.8% | 1.2% |
| Jiangsu | 6.8% | 5.7% | 6.2% | 5.1% | 5.7% | 4.0% | 5.7% | 4.0% | 5.2% | 2.4% | 4.8% | 1.2% |
| Zhejiang | 6.7% | 5.6% | 6.1% | 5.0% | 5.6% | 3.9% | 5.6% | 3.9% | 5.2% | 2.3% | 4.7% | 1.2% |
| Anhui | 8.6% | 7.1% | 7.8% | 6.4% | 7.1% | 5.0% | 7.1% | 5.0% | 6.5% | 3.0% | 5.9% | 1.5% |
| Fujian | 8.5% | 7.1% | 7.8% | 6.4% | 7.1% | 5.0% | 7.1% | 5.0% | 6.5% | 3.0% | 5.9% | 1.5% |
| Jiangxi | 7.4% | 6.2% | 6.8% | 5.6% | 6.2% | 4.3% | 6.2% | 4.3% | 5.7% | 2.6% | 5.2% | 1.3% |
| Shandong | 4.6% | 3.8% | 4.2% | 3.4% | 3.8% | 2.7% | 3.8% | 2.7% | 3.5% | 1.6% | 3.2% | 0.8% |
| Henan | 7.1% | 5.9% | 6.5% | 5.3% | 5.9% | 4.2% | 5.9% | 4.2% | 5.4% | 2.5% | 4.9% | 1.3% |
| Hubei | 8.2% | 6.8% | 7.5% | 6.1% | 6.8% | 4.8% | 6.8% | 4.8% | 6.3% | 2.9% | 5.7% | 1.4% |
| Hunan | 6.8% | 5.7% | 6.3% | 5.1% | 5.7% | 4.0% | 5.7% | 4.0% | 5.2% | 2.4% | 4.8% | 1.2% |
| Guangdong | 7.1% | 5.9% | 6.5% | 5.3% | 5.9% | 4.1% | 5.9% | 4.1% | 5.4% | 2.5% | 4.9% | 1.2% |
| Guangxi | 5.9% | 4.9% | 5.4% | 4.4% | 4.9% | 3.5% | 4.9% | 3.5% | 4.5% | 2.1% | 4.1% | 1.1% |
| Hainan | 6.5% | 5.4% | 5.9% | 4.9% | 5.4% | 3.8% | 5.4% | 3.8% | 5.0% | 2.3% | 4.5% | 1.1% |
| Chongqing | 8.5% | 7.1% | 7.8% | 6.4% | 7.1% | 5.0% | 7.1% | 5.0% | 6.5% | 3.0% | 5.9% | 1.5% |
| Sichuan | 7.1% | 6.0% | 6.6% | 5.4% | 6.0% | 4.2% | 6.0% | 4.2% | 5.5% | 2.5% | 5.0% | 1.3% |
| Guizhou | 9.3% | 7.8% | 8.6% | 7.0% | 7.8% | 5.5% | 7.8% | 5.5% | 7.2% | 3.3% | 6.5% | 1.7% |
| Yunnan | 8.7% | 7.2% | 8.0% | 6.5% | 7.2% | 5.1% | 7.2% | 5.1% | 6.6% | 3.1% | 6.0% | 1.5% |
| Shaanxi | 6.5% | 5.4% | 6.0% | 4.9% | 5.4% | 3.8% | 5.4% | 3.8% | 5.0% | 2.3% | 4.5% | 1.1% |
| Gansu | 5.2% | 4.3% | 4.7% | 3.9% | 4.3% | 3.0% | 4.3% | 3.0% | 4.0% | 1.8% | 3.6% | 0.9% |
| Qinghai | 5.2% | 4.4% | 4.8% | 3.9% | 4.4% | 3.1% | 4.4% | 3.1% | 4.0% | 1.9% | 3.7% | 0.9% |
| Ningxia | 5.7% | 4.8% | 5.3% | 4.3% | 4.8% | 3.4% | 4.8% | 3.4% | 4.4% | 2.0% | 4.0% | 1.0% |
| Xinjiang | 6.2% | 5.2% | 5.7% | 4.7% | 5.2% | 3.6% | 5.2% | 3.6% | 4.8% | 2.2% | 4.4% | 1.1% |
| China | 6.0% | 5.0% | 5.5% | 4.5% | 5.0% | 3.5% | 5.0% | 3.5% | 4.5% | 2.5% | 4.0% | 1.5% |

**Supplementary Table 4 | Future urbanization rate of each province.**

| | 2025 | 2030 | 2035 | 2040 | 2045 | 2050 | 2055 | 2060 |
|---|---|---|---|---|---|---|---|---|
| Beijing | 87% | 88% | 89% | 89% | 90% | 91% | 91% | 92% |
| Tianjin | 86% | 87% | 88% | 89% | 89% | 90% | 90% | 91% |
| Hebei | 60% | 63% | 65% | 67% | 70% | 73% | 75% | 78% |
| Shanxi | 60% | 62% | 63% | 65% | 67% | 69% | 71% | 74% |
| Inner Mongolia | 65% | 66% | 68% | 69% | 70% | 72% | 73% | 75% |
| Liaoning | 70% | 71% | 73% | 75% | 76% | 78% | 79% | 81% |
| Jilin | 60% | 61% | 62% | 63% | 65% | 66% | 67% | 69% |
| Heilongjiang | 63% | 65% | 67% | 69% | 71% | 73% | 74% | 75% |
| Shanghai | 89% | 89% | 90% | 90% | 90% | 91% | 91% | 92% |
| Jiangsu | 72% | 74% | 75% | 77% | 78% | 80% | 82% | 83% |
| Zhejiang | 72% | 74% | 75% | 77% | 79% | 80% | 82% | 84% |
| Anhui | 58% | 59% | 60% | 62% | 64% | 65% | 67% | 68% |
| Fujian | 68% | 70% | 72% | 73% | 75% | 77% | 78% | 80% |
| Jiangxi | 60% | 62% | 64% | 66% | 69% | 71% | 74% | 76% |
| Shandong | 64% | 65% | 67% | 69% | 71% | 73% | 75% | 77% |
| Henan | 56% | 58% | 59% | 61% | 63% | 64% | 66% | 68% |
| Hubei | 63% | 65% | 67% | 68% | 70% | 72% | 74% | 76% |
| Hunan | 60% | 62% | 65% | 67% | 70% | 73% | 76% | 79% |
| Guangdong | 72% | 73% | 74% | 75% | 76% | 77% | 78% | 79% |
| Guangxi | 53% | 55% | 57% | 60% | 62% | 64% | 66% | 69% |
| Hainan | 61% | 63% | 64% | 66% | 67% | 69% | 71% | 73% |
| Chongqing | 68% | 69% | 70% | 71% | 72% | 74% | 75% | 76% |
| Sichuan | 56% | 58% | 60% | 63% | 65% | 67% | 70% | 72% |
| Guizhou | 52% | 54% | 56% | 59% | 61% | 64% | 66% | 69% |
| Yunnan | 51% | 54% | 56% | 58% | 61% | 64% | 66% | 69% |
| Shaanxi | 62% | 64% | 66% | 69% | 71% | 73% | 76% | 78% |
| Gansu | 51% | 54% | 56% | 59% | 61% | 64% | 67% | 70% |
| Qinghai | 58% | 61% | 63% | 66% | 68% | 71% | 74% | 77% |
| Ningxia | 62% | 64% | 66% | 68% | 70% | 72% | 75% | 77% |
| Xinjiang | 54% | 57% | 59% | 61% | 64% | 66% | 69% | 72% |

**Supplementary Table 5 | Future secondary industrial share of each province.**

| | 2025 | 2030 | 2035 | 2040 | 2045 | 2050 | 2055 | 2060 |
|---|---|---|---|---|---|---|---|---|
| Beijing | 14% | 11% | 9% | 7% | 6% | 6% | 5% | 5% |
| Tianjin | 31% | 30% | 29% | 28% | 27% | 26% | 25% | 24% |
| Hebei | 40% | 39% | 38% | 38% | 37% | 37% | 36% | 35% |
| Shanxi | 37% | 35% | 34% | 33% | 32% | 30% | 29% | 28% |
| Inner Mongolia | 38% | 37% | 36% | 36% | 36% | 35% | 35% | 34% |
| Liaoning | 33% | 31% | 30% | 28% | 27% | 26% | 24% | 23% |
| Jilin | 31% | 28% | 25% | 23% | 20% | 17% | 14% | 11% |
| Heilongjiang | 23% | 21% | 20% | 19% | 18% | 17% | 16% | 15% |
| Shanghai | 23% | 21% | 20% | 19% | 17% | 16% | 15% | 14% |
| Jiangsu | 43% | 42% | 41% | 40% | 40% | 39% | 38% | 37% |
| Zhejiang | 40% | 39% | 38% | 37% | 36% | 36% | 35% | 34% |
| Anhui | 37% | 36% | 35% | 34% | 33% | 32% | 29% | 25% |
| Fujian | 42% | 39% | 36% | 32% | 29% | 26% | 22% | 19% |
| Jiangxi | 41% | 40% | 39% | 38% | 37% | 36% | 35% | 34% |
| Shandong | 38% | 37% | 36% | 35% | 34% | 34% | 33% | 32% |
| Henan | 41% | 39% | 39% | 38% | 37% | 36% | 36% | 35% |
| Hubei | 39% | 38% | 38% | 37% | 36% | 36% | 35% | 35% |
| Hunan | 35% | 34% | 33% | 32% | 31% | 30% | 29% | 28% |
| Guangdong | 39% | 38% | 38% | 37% | 37% | 36% | 35% | 35% |
| Guangxi | 33% | 33% | 32% | 31% | 31% | 30% | 29% | 29% |
| Hainan | 20% | 20% | 19% | 19% | 18% | 18% | 17% | 17% |
| Chongqing | 37% | 36% | 35% | 33% | 31% | 29% | 27% | 25% |
| Sichuan | 33% | 31% | 30% | 29% | 27% | 26% | 25% | 24% |
| Guizhou | 36% | 35% | 35% | 34% | 34% | 33% | 33% | 33% |
| Yunnan | 33% | 32% | 31% | 31% | 30% | 29% | 29% | 28% |
| Shaanxi | 41% | 40% | 40% | 39% | 39% | 38% | 37% | 37% |
| Gansu | 35% | 33% | 31% | 29% | 27% | 25% | 23% | 21% |
| Qinghai | 38% | 38% | 38% | 38% | 38% | 37% | 36% | 35% |
| Ningxia | 41% | 41% | 40% | 40% | 39% | 39% | 39% | 38% |
| Xinjiang | 31% | 30% | 29% | 27% | 26% | 25% | 24% | 23% |

**Supplementary Table 6 | Nonlinear regression results for Chinese provinces.** Note that the statistical tests used were two-sided and did not make adjustment for multiple comparisons. The panel data of each province used here includes 4 independent variables with a time span of 18 years.

| | ln*GDP* | ln*GDP*$^2$ | ln*GDP*$^3$ | ln*PV* | ln*UR* | ln*SI* | *a* | $R^2$ |
|---|---|---|---|---|---|---|---|---|
| Beijing | 0.035** | 0.017** | 0.012** | 0.955** | 0.890* | 0.202** | -4.716 | 0.79 |
| Tianjin | 0.298* | 0.149* | 0.099* | 3.330** | -2.569** | 1.567* | -18.243 | 0.83 |
| Hebei | 0.310 | 0.155 | 0.103 | 14.583 | -3.129 | 2.064 | -125.131 | 0.93 |
| Shanxi | 0.038 | 0.019 | 0.013 | 7.688* | -1.388* | 0.205 | -48.632 | 0.86 |
| Inner Mongolia | 0.435 | 0.217 | 0.145 | 4.049 | -1.427 | 2.104 | -35.839 | 0.92 |
| Liaoning | 0.827 | 0.413 | 0.276 | 3.792 | -1.445 | 1.372 | -45.421 | 0.75 |
| Jilin | 0.282** | 0.141** | 0.094** | 1.854* | -1.894 | 3.028 | -16.731 | 0.83 |
| Heilongjiang | 0.448 | 0.224 | 0.149 | 3.815* | 0.763 | 0.487 | -39.427 | 0.84 |
| Shanghai | 0.247 | 0.124 | 0.082 | 6.742 | 0.580** | 1.419*** | -58.906 | 0.79 |
| Jiangsu | 0.073 | 0.037 | 0.024 | 4.137* | -0.466 | 0.215 | -28.529 | 0.92 |
| Zhejiang | 0.140 | 0.070 | 0.047 | 2.512 | -0.425** | 0.322*** | -15.932 | 0.96 |
| Anhui | 0.092** | 0.046** | 0.031** | 11.719* | -0.178 | 2.132** | -102.891 | 0.93 |
| Fujian | 0.062 | 0.031 | 0.021 | 4.623 | -0.751** | 0.165 | -28.221 | 0.95 |
| Jiangxi | -0.050** | -0.025** | -0.017** | 5.070 | 0.323** | -0.390** | -32.281 | 0.94 |
| Shandong | 0.413*** | 0.206*** | 0.138*** | 13.619 | -2.555** | 2.190** | -126.028 | 0.92 |
| Henan | -0.009* | -0.005* | -0.003* | 5.606 | -0.477 | 0.814** | -42.690 | 0.73 |
| Hubei | 0.112** | 0.056** | 0.037** | 5.200*** | -0.559 | 1.445 | -42.150 | 0.88 |
| Hunan | 0.109* | 0.054* | 0.036* | 4.339*** | -0.698* | 0.645** | -31.912 | 0.85 |
| Guangdong | -0.050*** | -0.025*** | -0.017*** | 7.473* | -2.890** | 0.942 | -49.253 | 0.74 |
| Guangxi | -0.077** | -0.039** | -0.026** | 2.404* | 0.455** | -0.823** | -7.782 | 0.99 |
| Hainan | -0.010 | -0.005 | -0.003 | 2.520 | 0.008 | -0.767 | -6.778 | 0.90 |
| Chongqing | 0.149* | 0.075* | 0.050* | 3.885*** | -0.753** | 0.871 | -26.827 | 0.83 |
| Sichuan | 0.304 | 0.152 | 0.101 | 2.967 | -1.181** | 0.709*** | -24.471 | 0.94 |
| Guizhou | 0.084* | 0.042* | 0.028* | 1.714 | -0.191* | 0.280 | -7.279 | 0.87 |
| Yunnan | 0.029* | 0.015* | 0.010* | 2.535*** | 0.242 | -0.191** | -12.925 | 0.79 |
| Shaanxi | 0.190** | 0.095** | 0.063** | 7.033** | -1.308* | 0.693** | -51.230 | 0.97 |
| Gansu | 0.142 | 0.071 | 0.047 | 2.931* | -0.208*** | 0.059 | -17.167 | 0.90 |
| Qinghai | 0.231 | 0.115 | 0.077 | 1.707 | -0.190** | 0.374 | -8.625 | 0.85 |
| Ningxia | -0.190** | -0.095** | -0.063** | 6.894* | -0.075 | -1.255** | -26.444 | 0.92 |
| Xinjiang | -0.048 | -0.024 | -0.016 | 2.926 | -0.221 | -1.201 | -6.559 | 0.95 |

*, **, ***respectively represent p＜0.1, p＜0.05, p＜0.01

## Supplementary Figures

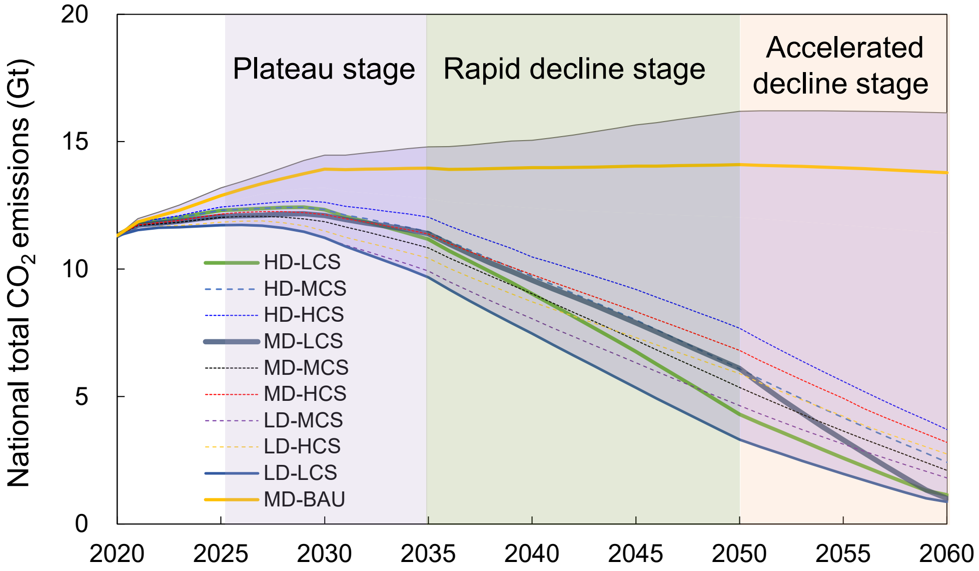


**Supplementary Fig. 1| The optimal carbon peak and carbon neutrality pathway for China.** C[3]IAM/NET model is used to investigate the carbon peak and carbon neutrality pathway for China by considering two types of uncertainties, including ① future product (or service) demand in energy-consuming sectors corresponding to different socio-economic development speeds, and ② the carbon sink potential which would determine the desired transition speed for energy system (fewer carbon sink in 2060, then a higher transition speed is required for the energy system, and vice versa). By integrating these two uncertainties, we designed ten scenarios for investigating the carbon peak and carbon neutral pathway for China, which are BAU, and a combination of three levels for the demand (high-demand [HD], medium-demand [MD], and low-demand [LD]) and three levels for carbon sink potential (high carbon sink with 3 billion tons available in 2060 [HCS], middle carbon sink with 2 billion tons available in 2060 [MCS], and low carbon sink with 1 billion tons in 2060 [LCS]). The pink ribbon in the figure is the uncertainty range for emissions. Note that the $CO_2$ emissions include fossil fuel related emissions and industrial process emissions. Fossil fuel related emissions corresponding to MD-LCS scenario is used as the constraint in the regional model analysis, because that ① according to the current planning of China, the medium GDP growth is more likely to occur, and ② according to Ref [29], 1 billion tons of carbon sink in 2060 is more possible for China.

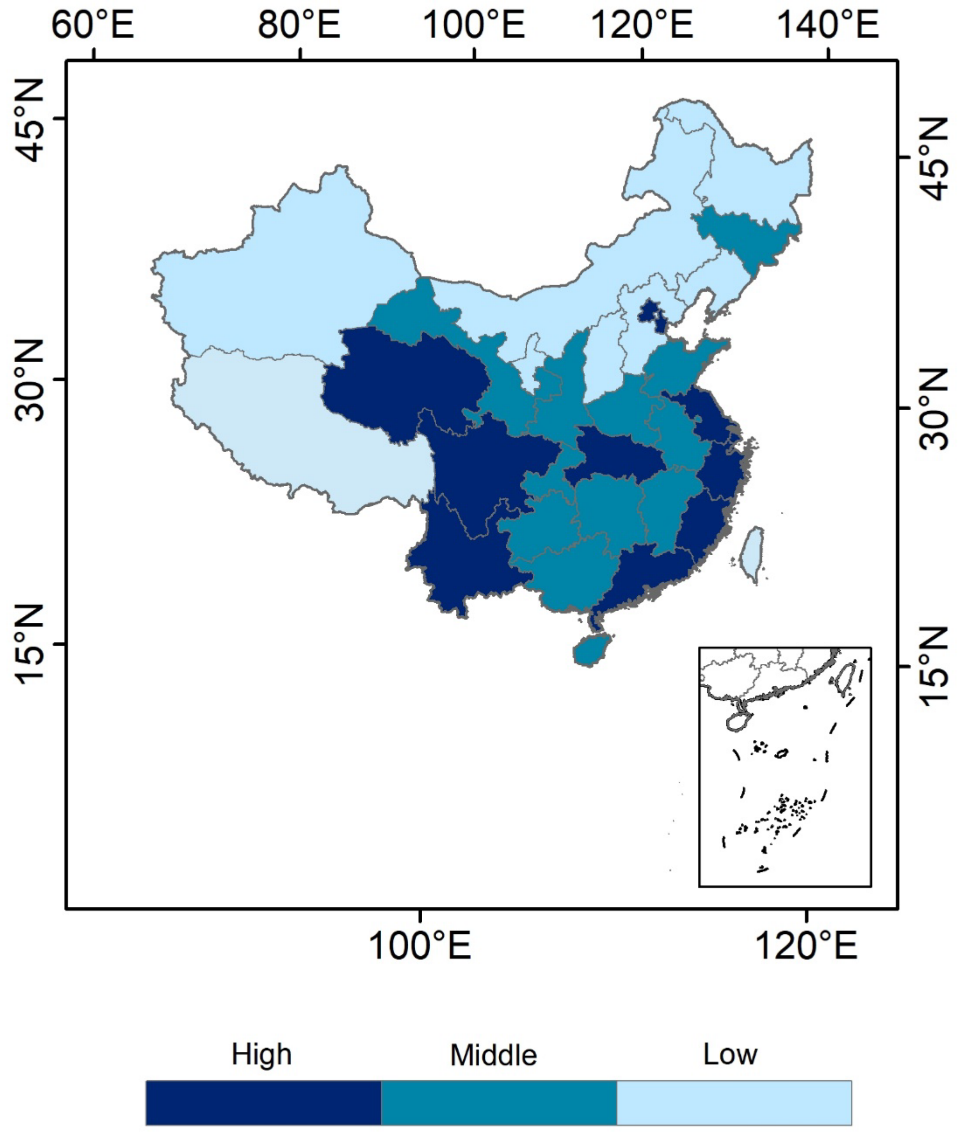


**Supplementary Fig. 2| Provinces grouped by the maturity index score.**

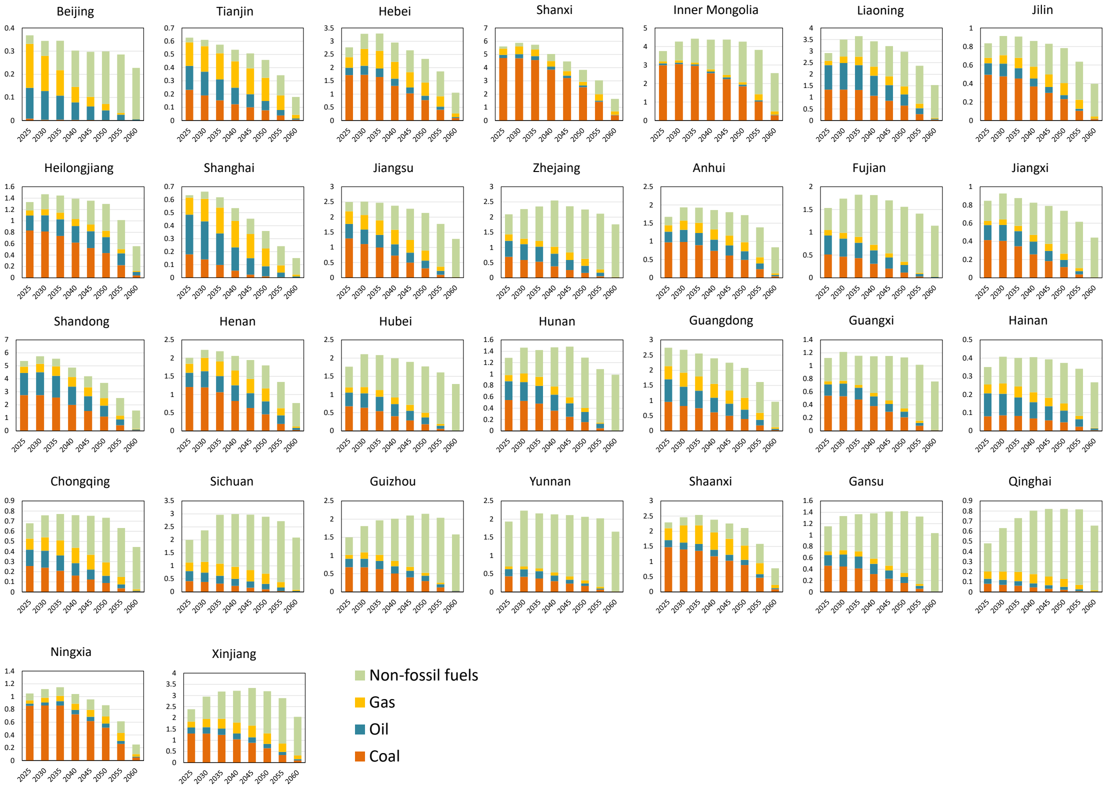


**Supplementary Fig. 3| Energy consumption of each province.**

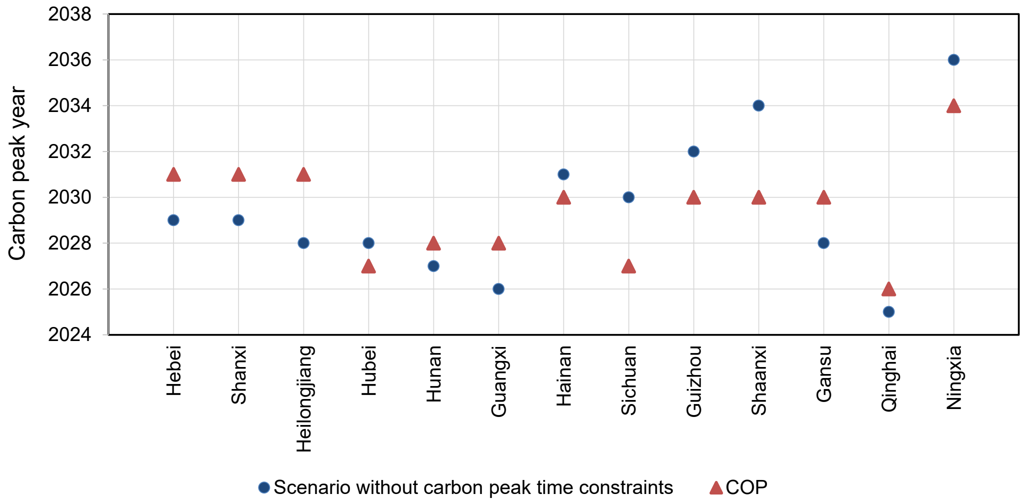


**Supplementary Fig. 4| The carbon peak years of provinces under the scenario of not setting carbon peak year constraints and COP scenario.** Note that only provinces with different carbon peak years under two scenarios are shown here.

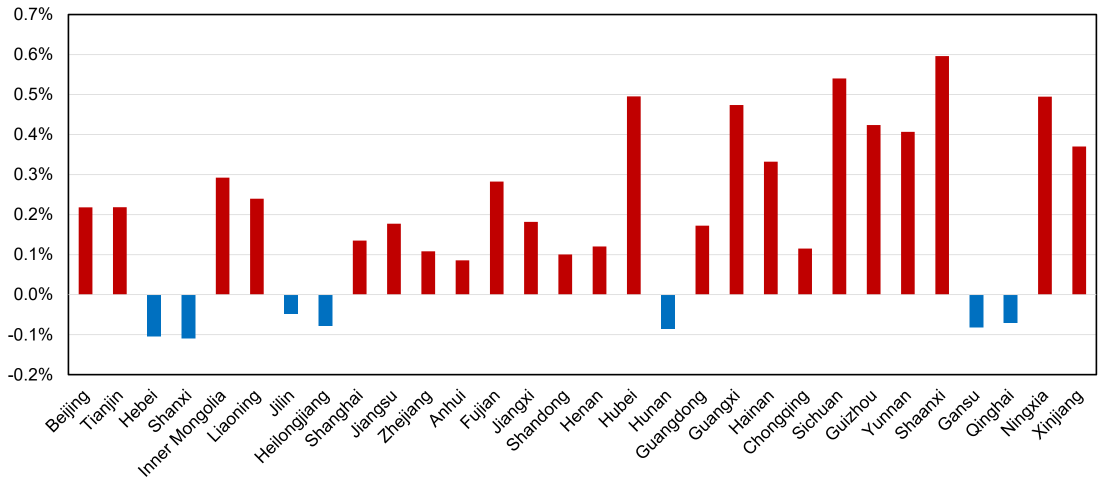


**Supplementary Fig. 5| Compared with the COP scenario, the economic benefits of each province under the scenario of not setting carbon peak year constraints.**

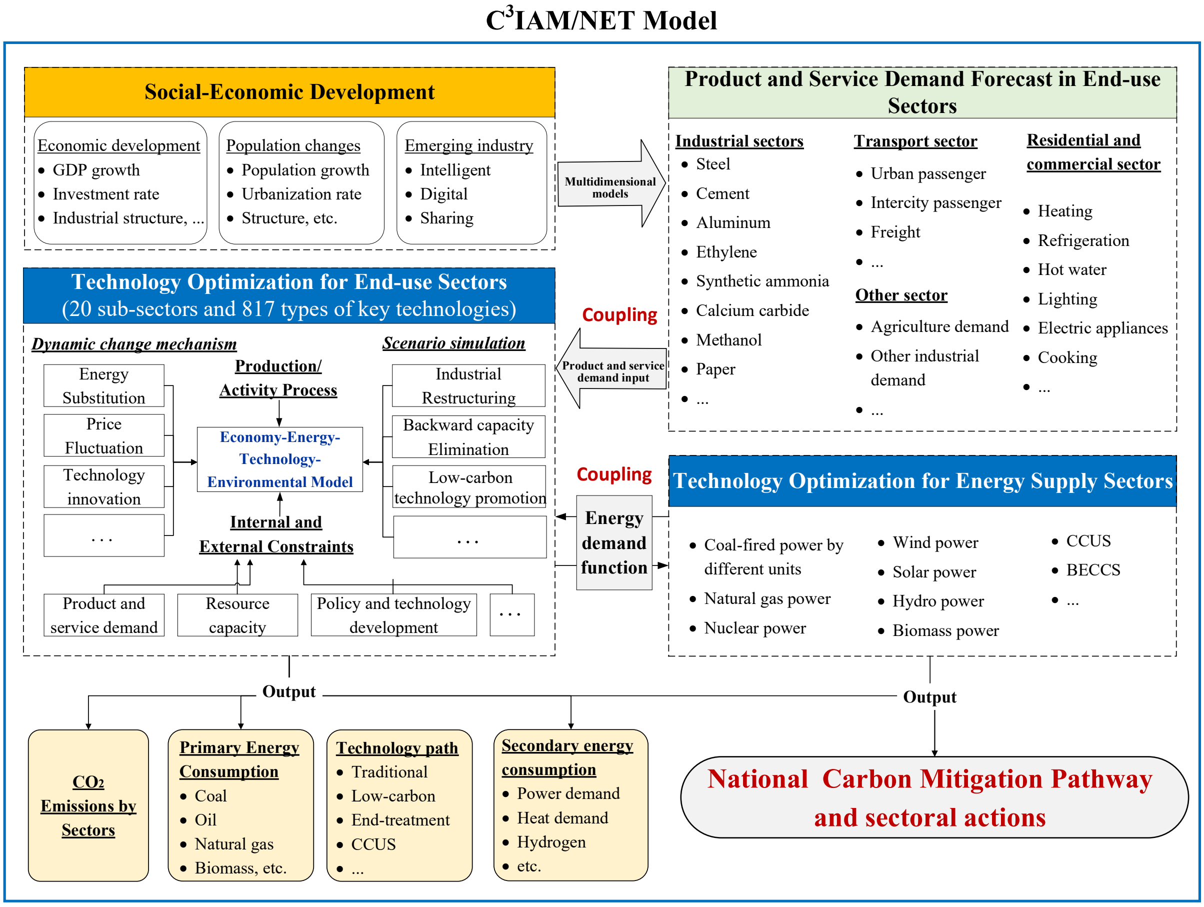


**Supplementary Fig .6 | Framework for C$^3$IAM/NET model.**

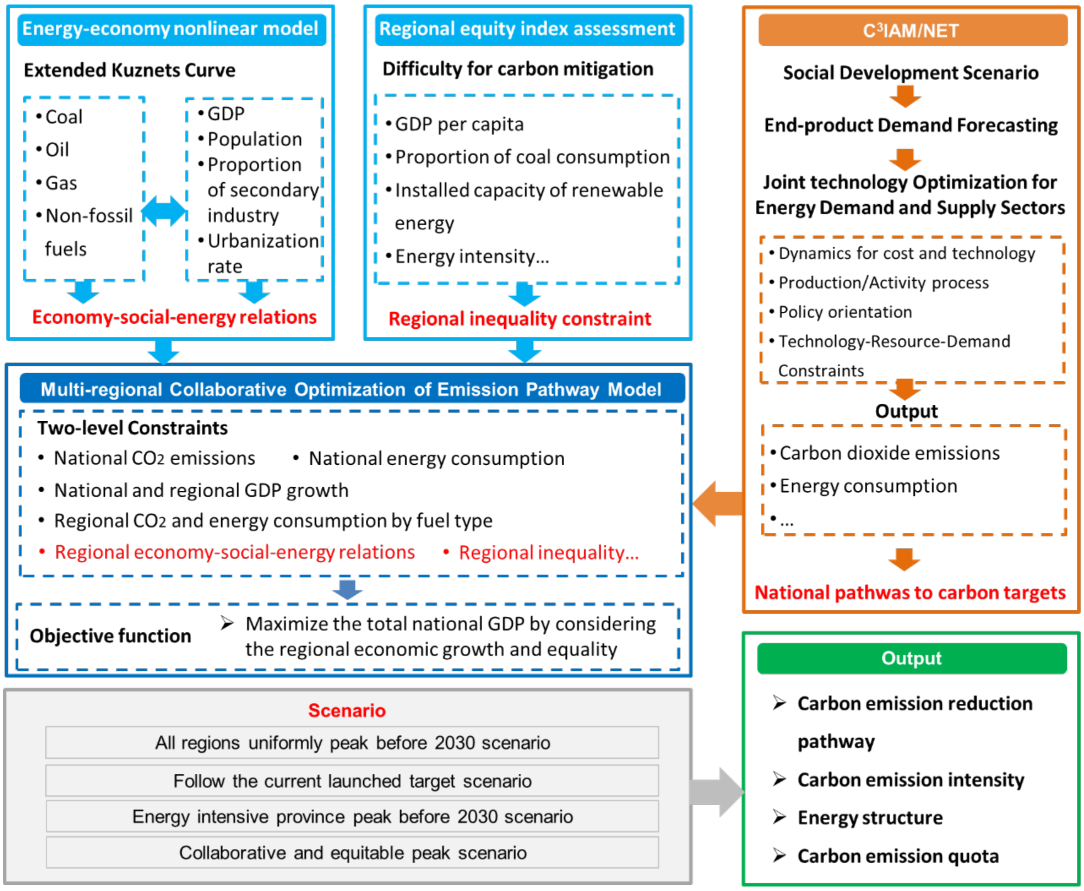


**Supplementary Fig .7 | Research framework for Mr. COEP model.**
