# Supplementary material for: Approaching national climate targets in China considering the challenge of regional inequality
Source: Nat Commun. 2023 Dec 15;14:8342. doi: 10.1038/s41467-023-44122-0 (PMC10724292; doi:10.1038/s41467-023-44122-0)
Supplement: Supplementary file 3 — Description of Additional Supplementary Files [file 41467_2023_44122_MOESM3_ESM.pdf]

Description of Additional Supplementary Files

File Name: Supplementary Data 1

Description: The data for GDP, population, second industrial proportion, urbanization rate for each provinces is list in Supplementary Data 1.
