# Supplementary material for: Approaching national climate targets in China considering the challenge of regional inequality
Source: Nat Commun. 2023 Dec 15;14:8342. doi: 10.1038/s41467-023-44122-0 (PMC10724292; doi:10.1038/s41467-023-44122-0)
Supplement: Supplementary file 5 — Reporting Summary [file 41467_2023_44122_MOESM5_ESM.pdf]

nature portfolio

Corresponding author(s): Biying Yu, Yi-Ming Wei, Lan-Cui Liu

Last updated by author(s): Nov 14, 2023

Reporting Summary

Nature Portfolio wishes to improve the reproducibility of the work that we publish. This form provides structure for consistency and transparency in reporting. For further information on Nature Portfolio policies, see our Editorial Policies and the Editorial Policy Checklist.

Statistics

For all statistical analyses, confirm that the following items are present in the figure legend, table legend, main text, or Methods section.

n/a	Confirmed	
☒	☐	The exact sample size (*n*) for each experimental group/condition, given as a discrete number and unit of measurement
☒	☐	A statement on whether measurements were taken from distinct samples or whether the same sample was measured repeatedly
☐	☒	The statistical test(s) used AND whether they are one- or two-sided<br>*Only common tests should be described solely by name; describe more complex techniques in the Methods section.*
☒	☐	A description of all covariates tested
☒	☐	A description of any assumptions or corrections, such as tests of normality and adjustment for multiple comparisons
☐	☒	A full description of the statistical parameters including central tendency (e.g. means) or other basic estimates (e.g. regression coefficient) AND variation (e.g. standard deviation) or associated estimates of uncertainty (e.g. confidence intervals)
☐	☒	For null hypothesis testing, the test statistic (e.g. *F*, *t*, *r*) with confidence intervals, effect sizes, degrees of freedom and *P* value noted<br>*Give P values as exact values whenever suitable.*
☒	☐	For Bayesian analysis, information on the choice of priors and Markov chain Monte Carlo settings
☒	☐	For hierarchical and complex designs, identification of the appropriate level for tests and full reporting of outcomes
☒	☐	Estimates of effect sizes (e.g. Cohen's *d*, Pearson's *r*), indicating how they were calculated

Our web collection on statistics for biologists contains articles on many of the points above.

Software and code

Policy information about availability of computer code

Data collection	The data were from the iNEMS database of the Center for Energy and Environmental Policy Research of Beijing Institute of Technology, China National Bureau of Statistics, Chinese provincial statistical yearbooks, official policy documents launched by Chinese provincial governments, and necessary references (listed in manuscript) .
Data analysis	IBM SPSS Statistics 26.0.0 is used for nonlinear regression; GAMS 24.8.3 is used fo solve the multi-regional collaborative emission reduction pathway optimization model.

For manuscripts utilizing custom algorithms or software that are central to the research but not yet described in published literature, software must be made available to editors and reviewers. We strongly encourage code deposition in a community repository (e.g. GitHub). See the Nature Portfolio guidelines for submitting code & software for further information.

Data

Policy information about availability of data

All manuscripts must include a data availability statement. This statement should provide the following information, where applicable:

- Accession codes, unique identifiers, or web links for publicly available datasets
- A description of any restrictions on data availability
- For clinical datasets or third party data, please ensure that the statement adheres to our policy

All kinds of provincial energy consumption data were from the iNEMS database of the Center for Energy and Environmental Policy Research of Beijing Institute of

Technology53. National greenhouse gas inventory data were used for various energy emission factors, including 2.66 tons of CO_2/standard coal for coal, 1.73 tons of CO_2/standard coal for oil products, and 1.56 tons of CO_2/standard coal for natural gas. The historical GDP data for each province were from the National Bureau of Statistics, and the forecast data were from the high-speed and low-speed scenario data in the ref34 (See Supplementary Table 1 for specific settings). The GDP data were uniformly converted into GDP values with 2020 as the constant price through the GDP index. This study sets the upper and lower limits of GDP for each province in future years according to the deviation degree from the national GDP growth rate (See Supplementary Table 3). The historical population data for each province is from the National Bureau of Statistics54, and the future population forecast is from ref50. The energy consumption per unit output value and the proportion of secondary industry in each province were derived from the National Bureau of Statistics and provincial statistical yearbooks. The historical and future urbanization rate data for each province are from the National Bureau of Statistics and ref48,51,52. Please see the indicators in Supplementary Data 1 file. The future settings of urbanization and secondary industrial share can be seen in Supplementary Table 4 and Table 5. The planning goals for each province, such as the year of carbon peak, energy intensity, coal proportion, non-fossil energy proportion, and other data, were collected from the official policy documents launched by provincial governments (Supplementary Table 2).

Research involving human participants, their data, or biological material

Policy information about studies with human participants or human data. See also policy information about sex, gender (identity/presentation), and sexual orientation and race, ethnicity and racism.

Reporting on sex and gender	Not applicable.
Reporting on race, ethnicity, or other socially relevant groupings	Not applicable.
Population characteristics	Not applicable.
Recruitment	Not applicable.
Ethics oversight	Not applicable.

Note that full information on the approval of the study protocol must also be provided in the manuscript.

Field-specific reporting

Please select the one below that is the best fit for your research. If you are not sure, read the appropriate sections before making your selection.

☐ Life sciences ☐ Behavioural & social sciences ☒ Ecological, evolutionary & environmental sciences

For a reference copy of the document with all sections, see nature.com/documents/nr-reporting-summary-flat.pdf

Ecological, evolutionary & environmental sciences study design

All studies must disclose on these points even when the disclosure is negative.

Study description	We propose economically optimal strategies that can achieve national climate targets and ensure the greatest local and national benefits as well as regional equality.
Research sample	We selected all provinces in China where data can be obtained for empirical research, specifically 30 provinces except Tibet, Taiwan, Hong Kong and Macao.
Sampling strategy	We selected variables with significant impact and extended the time span of these variables as much as possible.
Data collection	The data were from the INEMS database of the Center for Energy and Environmental Policy Research of Beijing Institute of technology , China National Bureau of Statistics , Chinese provincial statistical yearbooks , official policy documents launched by Chinese provincial governments , and necessary references (listed in manuscript). Zihao Zhao recorded the data.
Timing and spatial scale	Timing scale : 2020-2060. Spatial scale : 30 Chinese provinces except Tibet , Hong Kong , Macao , and Taiwan
Data exclusions	No data were excluded from the analyses.
Reproducibility	This study did not include any experiment. Regarding the method, this study solved the optimization problem using GAMS 24.8.3 and CONOPT Optimizer in the simulation section
Randomization	Randomization is not relevant to this study , because this study only used the statistical raw data for projection and optimization.
Blinding	Blinding is not relevant to this study , because there is no experiment in this study.

Did the study involve field work? ☐ Yes ☒ No

Reporting for specific materials, systems and methods

We require information from authors about some types of materials, experimental systems and methods used in many studies. Here, indicate whether each material, system or method listed is relevant to your study. If you are not sure if a list item applies to your research, read the appropriate section before selecting a response.

Materials & experimental systems

n/a	Involved in the study
☒	☐ Antibodies
☒	☐ Eukaryotic cell lines
☒	☐ Palaeontology and archaeology
☒	☐ Animals and other organisms
☒	☐ Clinical data
☒	☐ Dual use research of concern
☒	☐ Plants

Methods

n/a	Involved in the study
☒	☐ ChIP-seq
☒	☐ Flow cytometry
☒	☐ MRI-based neuroimaging

Plants

Seed stocks

Not applicable.

Novel plant genotypes

Not applicable.

Authentication

Not applicable.
